# MMP13 is a critical target gene during the progression of osteoarthritis

**DOI:** 10.1186/ar4133

**Published:** 2013-01-08

**Authors:** Meina Wang, Erik R Sampson, Hongting Jin, Jia Li, Qiao H Ke, Hee-Jeong Im, Di Chen

**Affiliations:** 1 Department of Orthopedics and Rehabilitation, Center for Musculoskeletal Research, University of Rochester Medical Center, Rochester, NY 14642, USA; 2 Current address: Section of Endocrinology, Department of Internal Medicine, Yale University School of Medicine, 333 Cedar St., PO Box 208020, New Haven, CT 06520, USA; 3 Current address: Molecular and Cellular Pharmacology, Abbott, 100 Research Drive, Worcester, MA 01605, USA; 4 Institute of Orthopedics and Traumatology, Zhejiang Chinese Medical University, 548 Binwen Road, Binjiang District, Hangzhou 310053, Zhejiang Province, China; 5 Department of Biochemistry, Rush University Medical Center, Chicago, 1735 West Harrison Street, IL 60612, USA; 6 Liaoning University of Traditional Chinese Medicine, 79 East Chongshan Road, Huanggu District, Shenyang 110847, Liaoning Province, China

## Abstract

**Introduction:**

Osteoarthritis (OA) is a degenerative joint disease affecting a large population of people. The mechanism of this highly prevalent disease is not fully understood. Currently there is no effective disease-modifying treatment for OA. The purpose of this study was two-fold: 1) to investigate the role of MMP13 in the development of OA; and 2) to evaluate the efficacy of the MMP13 inhibitor CL82198 as a pharmacologic treatment for preventing OA progression.

**Methods:**

To investigate the role of the endogenous *Mmp13 *gene in OA development, tamoxifen was administered to two-week-old *Col2CreER;Mmp13^fx/fx ^*(*Mmp13^Col2ER^*) and Cre-negative control mice for five days. OA was induced by meniscal-ligamentous injury (MLI) when the mice were 10 weeks old and MLI or sham-operated joints were harvested 4, 8, 12, or 16 weeks after surgery. To evaluate the efficacy of CL82198, MLI surgery was performed on 10-week-old wild type mice. CL82198 or saline was administered to the mice daily beginning immediately after the surgery for up to 16 weeks. The joint tissues collected from both experiments were evaluated by cartilage grading, histology/histomorphometry, immunohistochemistry (IHC), and terminal deoxynucleotidyl transferase dUTP nick end labeling (TUNEL) staining. The ability of CL82198 to inhibit MMP13 activity *in vitro *was confirmed by ELISA.

**Results:**

The OA progression was decelerated in *Mmp13^Col2ER ^*mice 8, 12, and 16 weeks post-surgery. Cartilage grading by blinded observers confirmed decreased articular cartilage degeneration in *Mmp13^Col2ER ^*mice at 8, 12 and 16 weeks compared to Cre-negative mice. Histomorphometric analysis demonstrated that *Mmp13^Col2ER ^*mice had a higher articular cartilage area and thickness at 12 and 16 weeks post-surgery compared to the control mice. Results of IHC revealed greater type II collagen and proteoglycan expression in *Mmp13^Col2ER ^*mice. Chondrocyte apoptosis, as determined by TUNEL staining, was higher in control mice compared to *Mmp13^Col2ER ^*mice. CL82198 inhibited MMP13 activity in conditioned media from vehicle (> 85%) or bone morphogenetic protein 2 (BMP2)-treated (> 90%) primary murine sternal chondrocytes. Intraperitoneal injection of CL82198 decelerated MLI-induced OA progression, increased type II collagen and proteoglycan levels, and inhibited chondrocyte apoptosis compared to saline treatment as determined by OA grading, histology, histomorphometry, IHC, and TUNEL staining, respectively.

**Conclusions:**

*Mmp13 *is critical for OA progression and pharmacologic inhibition of MMP13 is an effective strategy to decelerate articular cartilage loss in a murine model of injury-induced knee OA.

## Introduction

Osteoarthritis (OA) is a degenerative joint disease and is a major cause of disability. Currently, there is no treatment capable of altering its progression. The major pathological characteristics of OA include progressive loss of articular cartilage, osteophyte formation, and changes in peri-articular and subchondral bone [1,2]. The articular cartilage receives most of the attention in OA studies because the primary pathologic feature seen in OA is gross articular cartilage damage.

Matrix metalloproteinase (MMP) 13 is a major enzyme that targets cartilage for degradation. Compared to other MMPs, the expression of MMP13 is more restricted to connective tissue [3]. It not only targets type II collagen in cartilage for degradation, but also degrades proteoglycan, types IV and type IX collagen, osteonectin and perlecan in cartilage [4]. Clinical investigation revealed that patients with articular cartilage destruction have high MMP13 expression [5], suggesting that increased MMP13 may be associated with cartilage degradation. Studies have also shown that *Mmp13*-overexpressing transgenic mice develop a spontaneous OA-like articular cartilage destruction phenotype [6]. The ADAMTS (a disintegrin and metalloproteinase with thrombospondin motifs) family of aggrecanases also contributes to proteoglycan/aggrecan depletion and are associated with cartilage degradation during OA. ADAMTS4 and 5 were identified as the major aggrecanases during OA development. Deletion of the *Adamts5 *gene or double knockout of *Adamts4 *and *Adamts5 *prevented cartilage degradation in a surgically induced murine knee OA model [7,8]. These findings indicate that catabolic enzymes play a significant role in OA progression and support the development of therapies targeting these enzymes as a strategy to decelerate articular cartilage degradation.

Meniscal injuries are among the most common causes of post-traumatic OA in humans. The meniscus is a C-shaped cartilage that functions as a shock-absorbing, load bearing, stability enhancing, and lubricating cushion in the knee joint. Studies show that loss of meniscus integrity and function leads to OA in humans [9,10]. The meniscal-ligamentous injury (MLI)-induced murine OA model was initially developed by Clements *et al. *[11] and this injury model has been further modified and developed in recent studies [12]. This mouse model also permits study of the development of trauma-induced OA in defined genetic backgrounds. In this model, the ligation of the medial collateral ligament coupled with disruption of the meniscus from its anterior-medial attachment induces reproducible OA development over a three-month period [12]. In the present studies, we will determine the role of MMP13 in MLI-induced OA progression. We will use *Col2CreER;Mmp13^fx/fx ^*(*Mmp13^Col2ER^*) mice to block *Mmp13 *expression in chondrocytes and use MMP13 inhibitors to inhibit MMP13 activity.

## Methods

### Meniscal/ligamentous injury-induced osteoarthritis model and treatment of MMP13 inhibitor

Wild type C57BL/6J and *Mmp13*^fx/fx ^mice [13] were obtained from Jackson Laboratories (Bar Harbor, ME, USA). *Col2CreER *transgenic mice [14] were crossed with *Mmp13*^fx/fx ^mice to generate *Col2CreER;Mmp13*^fx/fx ^mice (referred to hereafter as *Mmp13^Col2ER ^*mice) (Table 1). Tamoxifen (Sigma, St. Louis, MO, USA) was administered to two-week-old *Mmp13^Col2ER ^*mice and littermate controls by intraperitoneal (i.p.) injection (1 mg/10 g body weight) for five days. MLI surgery was performed to induce knee OA [12] in 10-week-old *Mmp13^Col2ER ^*and Cre-negative control mice. Details regarding MLI surgery were previously described [12]. The surgery was performed on the right hind limbs as follows: 1) following anesthesia, making a 5 mm parapatellar; 2) identifying and transecting the medial collateral ligament with a 25 gauge needle; 3) applying valgus stress to the knee to confirm disruption and provide access to the meniscus; 4) detaching and partially removing the anterior horn of the medial meniscus; 5) closing of the wound with 4.0 nylon sutures applied in an interrupted pattern. The left hind limb was used as a control. The left hind limb was opened and the structures of the knee were exposed and then the skin incision closed without manipulating the joint tissue. Pre- and post-surgery, mice were provided analgesia (2.5 mg/kg banamine, i.p. injection) every 24 hours for 72 hours and the sutures were removed after 10 days. Both left and right knee joints were harvested, processed, sectioned and stained 4, 8, 12, and 16 weeks post-surgery (*n *= 5 in each group). MLI and sham surgeries were also performed on 10-week-old C57BL/6J mice (*n *= 5 in each group). The MMP13 inhibitor CL82198 (Tocris Bioscience, Minneapolis, MN, USA) was administered to wild type mice beginning one day after surgery by i.p. injection every other day for up to 16 weeks at doses of 1, 5, 10 mg/kg body weight. Normal saline was used as a control. Knee joints were collected, sectioned and stained 12 weeks post-surgery (*n *= 5 in each group). All protocols were approved by the Institutional Animal Care and Use Committee (IACUC) at the University of Rochester.

**Table 1:** Breeding of *Col2CreER;Mmp13^fx/fx ^*mice (*Mmp13^Col2ER^*).

Breeding	Desired progeny
a) *Col2CreER *x *Mmp13^fx/fx^*	a) *Col2CreER;Mmp13^fx/wt^*
b) *Col2CreER;Mmp13^fx/wt ^*x *Mmp13^fx/fx^*	b) *Col2CreER;Mmp13^fx/fx^*
c) *Col2CreER;Mmp13^fx/fx ^*x *Mmp13^fx/fx^*	c) *Col2CreER;Mmp13^fx/fx ^*and *Mmp13^fx/fx^*

### Histology and histomorphometry

Knee joints from each group were harvested and prepared for sectioning and analysis. Samples were fixed in 10% neutral buffered formalin (VWR, Radnor, PA, USA) for three days, then decalcified with formic acid (Decal Chemical Corp., Suffern, NY, USA) for seven days. After neutralizing with Cal-arrest (Decal Chemical Corp.), samples were processed and embedded in paraffin. Three μm thick mid-saggital sections at three different levels (50 μm apart) were cut from the medial compartment of the joints. The sections were stained with Alcian blue/H&E (AB/H&E) and safranin O/Fast Green (SO/FG). Histomorphometric measurements were performed using OsteoMeasure software (OsteoMetrics, Inc., Atlanta, GA, USA). AB/H&E- or SO/FG-stained areas were outlined on projected images of each histologic section to determine articular cartilage area and thickness.

### Immunohistochemistry and TUNEL staining

Three μm thick paraffin sections were baked at 60°C overnight. Slides were then deparaffinized, rehydrated, and digested with pepsin. DAKO endogenous blocking reagent (Dako S2003, Carpinteria, CA, USA) was then used to quench endogenous peroxidase for 10 minutes. Normal horse serum or normal goat serum (Vector S-2000, Burlingame, CA, USA) was used to block non-specific binding sites for 20 minutes. Collagen II or collagen X primary antibodies (Thermo Scientific, Rockford, IL, USA) were added and the slides were incubated at 4°C overnight. Secondary biotinylated horse anti-mouse antibody (Vector BA-2000) or goat anti-mouse antibody (Vector BA-9200) was added for 30 minutes on the second day, followed by incubation with streptavidin (Pierce 21130, Rockford, IL, USA) for 30 minutes. Positive staining was detected by Romulin AEC Chromagen (Biocare Medical RAEC810L, Concord, CA, USA). To detect chondrocyte apoptosis after meniscal surgery, terminal deoxynucleotidyl transferase dUTP nick end labeling (TUNEL) staining was performed using a kit based on the manufacturer's instruction (Promega, Fitchburg, WI, USA).

### Grading of cartilage structure

Tissue sections were stained with Alcian blue/Orange G and graded by two blinded observers based on the scoring system developed by Chambers *et al. *[7]. In brief, each section was assigned a grade as follows: 0 = normal cartilage, that is, lack of superficial zone fibrillation or clefting; 1 = mild superficial fibrillation; 2 = fibrillation and/or clefting extending below the superficial zone; 3 = mild (< 20%) loss of articular cartilage; 4 = moderate (20% to 80%) loss of articular cartilage; 5 = focal loss of cartilage to the subchondral bone (eburnation); 6 = severe (> 80%) loss of articular cartilage. The progression of OA in different groups was evaluated using this method. Three slides of each sample were analyzed and five mice were used in each group.

### MMP13 activity assay

The ability of CL82198 to inhibit MMP13 activity *in vitro *was determined using the SensoLyte 520 MMP13 Assay Kit (AnaSpec, Fremont, CA, USA). Five ng of MMP13 and 10 μg/mL of CL82198, or control substrate were added into the MMP13 Assay Kit. MMP13 activity was detected following the manufacturer's instructions. Primary sternal chondrocytes were isolated from three-day-old wild-type (WT) pups as previously described [15]. Cells were plated in 12-well-plates and treated with bone morphogenetic protein 2 (BMP-2) (100 ng/ml) alone or BMP-2 with the MMP-13 inhibitor CL82198 (1, 5 and 10 μM) for 60 hours. The culture media were collected and MMP13 activity was determined using the SensoLyte 520 MMP13 Assay Kit (AnaSpec) following the manufacturer's instructions.

### Statistical analysis

Results of all quantitative assays involving multiple doses and time points were analyzed using analysis of variance (ANOVA) followed by Dunnett's test. For experiments comparing two groups, unpaired student's *t*-test was applied. *P *< 0.05 was considered to be a significant difference.

## Results

### OA progression was decelerated in *Mmp13^Col2ER ^*mice

**Figure 1:**
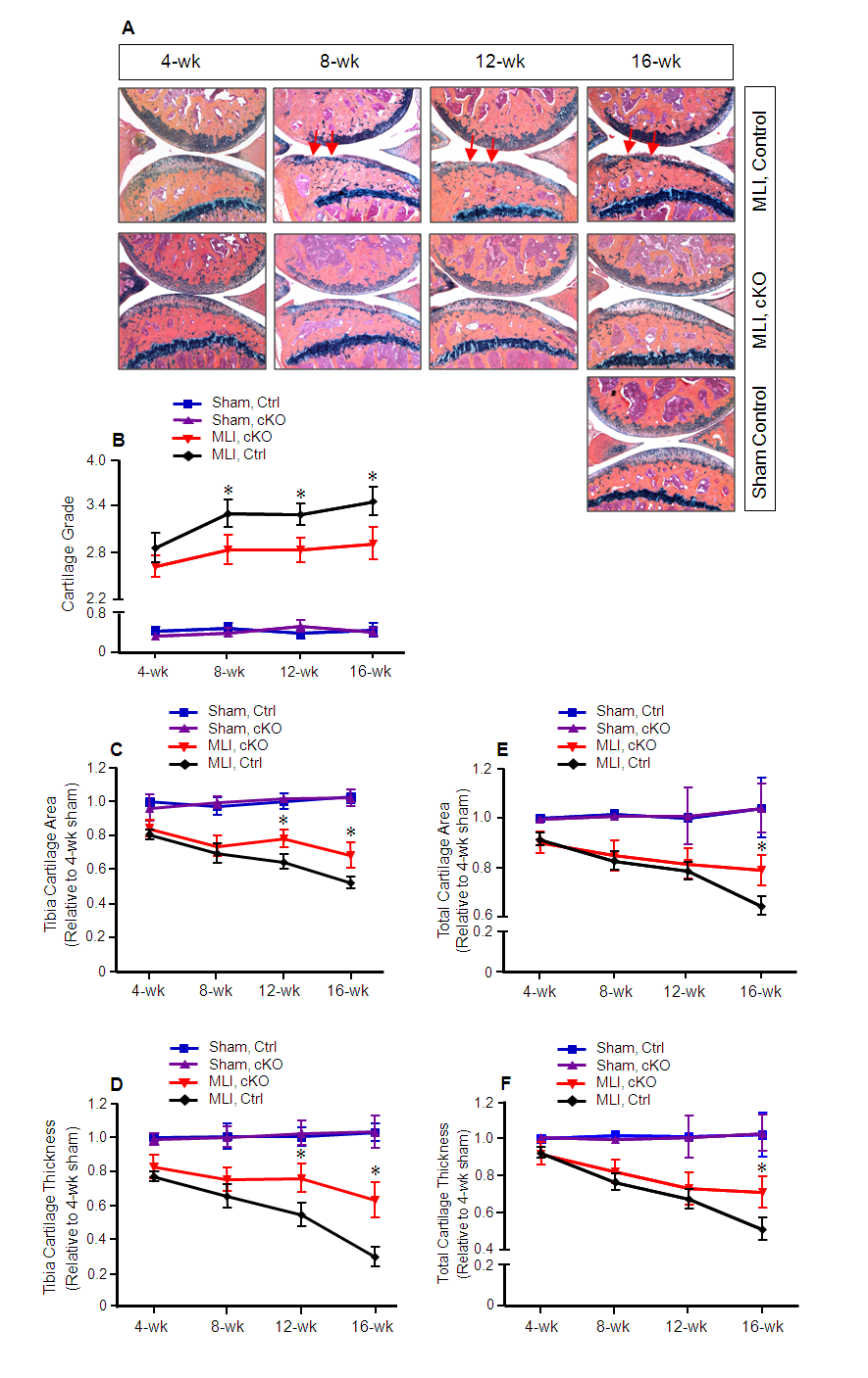
**Decelerated osteoarthritis progression in *Mmp13^Col2ER ^*mice**. Tamoxifen was administered when matrix metalloproteinase (MMP13) conditional knockout (cKO) mice (*Mmp13^Col2ER^*) and Cre-negative control mice were two weeks old (1 mg/10 g body weight, i.p., daily for five days). Meniscal-ligamentous injury (MLI)-induced osteoarthritis (OA) surgery was performed on the right hind-limbs when the mice were 10 weeks old. The left hind-limbs were used as sham controls. (**A**) Knee joint samples were harvested 4, 8, 12, or 16 weeks post-surgery and Alcian blue/Hematoxylin/Orange G staining was performed. Histological results showed decreased articular cartilage degradation in *Mmp13^Col2ER ^*mice 8, 12 and 16 weeks post-surgery. (**B**) Histological grading by blinded observers confirmed decreased articular cartilage degradation in *Mmp13^Col2ER ^*mice at 8, 12, and 16 weeks compared to control Cre-negative mice (*P < 0.05). (**C**) Tibia articular cartilage area was quantified by tracing the Alcian blue-positive area in the proximal tibia. There was no significant difference 4 and 8 weeks post-surgery in *Mmp13^Col2ER ^*MLI mice versus control MLI mice. The tibia cartilage area was increased 21% in *Mmp13^Col2ER ^*MLI mice compared to control MLI mice 12 weeks post-surgery (*P < 0.05) and increased 31% 16 weeks post-surgery (*P < 0.05). (**D**) Tibia thickness was quantified by tracing the Alcian blue-positive thickness in the center of the tibial plateau. There was no significant difference in tibial cartilage thickness at 4 or 8 weeks post-surgery. Tibia cartilage thickness was increased 29% in *Mmp13^Col2ER ^*MLI mice compared to control MLI mice 12 weeks post-surgery (*P < 0.05) and increased 50% 16 weeks post-surgery (*P < 0.01). (**E**) Total articular cartilage area was quantified by tracing the Alcian blue-positive area in both the proximal tibia and distal femur. No significant differences were detected 4, 8, and 12 weeks post-surgery. Total cartilage area increased 18% in *Mmp13^Col2ER ^*MLI mice compared to control MLI mice 16 weeks post-surgery (*P < 0.01). (**F**) Total cartilage thickness was quantified by tracing the Alcian blue-positive thickness in the center of the proximal tibia and distal femur. No significant differences were detected at 4, 8, and 12 weeks post-surgery. Total cartilage thickness increased 39% in *Mmp13^Col2ER ^*MLI mice compared to control MLI mice 16 weeks post-surgery (*P < 0.01).

To investigate if *Mmp13 *deletion could prevent or decelerate MLI-induced OA, we crossed *Col2CreER *transgenic mice [14,16] with *Mmp13*^fx/fx ^mice [13] to generate *Col2CreER;Mmp13*^fx/fx ^mice (*Mmp13^Col2ER^*) (Table 1). Deletion of the *Mmp13 *gene in chondrocytes had no significant effect on articular and growth plate chondrocyte morphology [see Additional file 1]. Tamoxifen was administered (i.p., five days) to two-week-old *Mmp13^Col2ER ^*mice and had no effect on articular and growth plate cartilage [see Additional file 1]. MLI surgery was performed when the mice were 10-weeks-old to induce OA. Knee joints were harvested 4, 8, 12, and 16 weeks post-surgery (*n *= 5 in each group). Histology showed that four weeks post-MLI surgery, articular cartilage was nearly normal in both *Mmp13^Col2ER ^*and Cre-negative control groups (Figure 1A). OA-like fibrillation, clefting and cartilage loss down to the tidemark appeared 8 weeks post-surgery and worsened at the 12- and 16-week time points in Cre-negative control mice. In *Mmp13^Col2ER ^*mice, however, there was markedly less articular cartilage excavation, especially at the 12- and 16-week time points (Figure 1A). OA grading likewise revealed significantly reduced cartilage degeneration at 8, 12 and 16 weeks post-surgery in *Mmp13^Col2ER ^*mice compared to the control group (Figure 1B). To quantify OA progression, we performed histomorphometry using the OsteoMeasure system. The results showed that articular cartilage area and articular cartilage thickness at proximal tibiae, total cartilage area, and total cartilage thickness progressively decreased at 8, 12, and 16 weeks post-surgery (Figure 1C-F), but this progression was decelerated in *Mmp13^Col2ER ^*mice compared to the control group. Articular cartilage area and articular cartilage thickness at proximal tibiae were significantly greater at 12 and 16 weeks post-surgery in *Mmp13^Col2ER ^*groups (Figure 1C-D). Total cartilage area was significantly different at 16 weeks post-surgery (Figure 1E), and total cartilage thickness was significantly different at 8, 12, and 16 weeks post-surgery (Figure 1F). Articular cartilage area and thickness at distal femora had no significant changes at any time point post-surgery.

#### Col2 and proteoglycan loss and chondrocyte apoptosis are reduced in *Mmp13^Col2ER ^*mice

**Figure 2:**
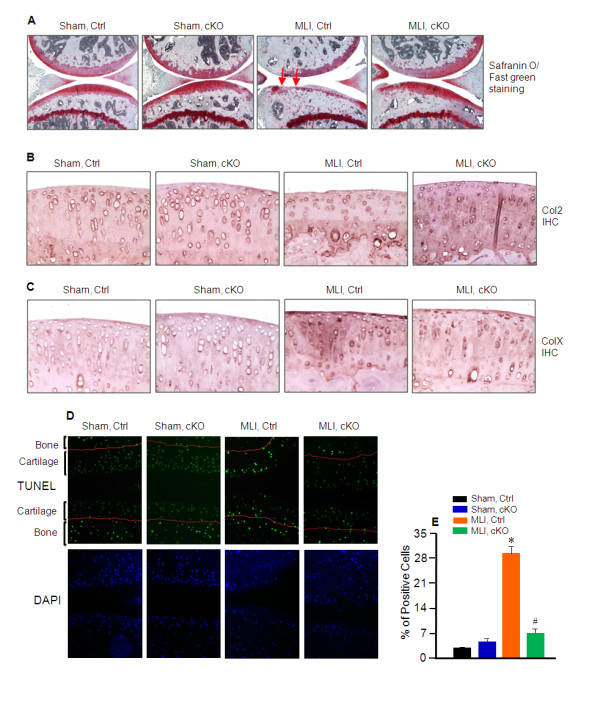
**Decelerated osteoarthritis progression in *Mmp-13^Col2ER ^*mice**. Tamoxifen was administered when matrix metalloproteinase (MMP13) conditional knockout (cKO) mice (*Mmp13^Col2ER^*) and Cre-negative control mice were two-weeks-old (1 mg/10 g body weight, intraperitoneal injection, daily for five days). Meniscal-ligamentous injury (MLI) surgery was performed when the mice were 10-weeks-old. Knee joints were harvested eight weeks post-surgery. (**A**) Safranin O/Fast Green staining was performed to evaluate proteoglycan content. Proteoglycan loss following MLI was reduced in *Mmp13^Col2ER ^*mice. **(B) **Type II collagen (Col2) loss following MLI was reduced in *Mmp13^Col2ER ^*mice. Immunohistochemistry (IHC) of Col2 was performed to assess its protein level. (**C**) IHC of ColX was performed to assess its protein level. Type X collagen (ColX) induction following MLI was reduced in *Mmp13^Col2ER ^*mice. (**D**) Terminal deoxynucleotidyl transferase dUTP nick end labeling (TUNEL) staining (in green) and 4',6-diamidino-2-phenylindole (DAPI) nuclear counterstaining (in blue) were performed to assess chondrocyte apoptosis. Chondrocyte apoptosis following MLI was reduced in *Mmp13^Col2ER ^*mice. (**E**) Quantification of TUNEL staining positive cells was performed to assess chondrocyte apoptosis.

Safranin O/Fast green (SO/FG) staining and IHC of Col2 and ColX were performed on both *Mmp13^Col2ER ^*and control mice eight weeks post-surgery. The results showed greater proteoglycan and Col2 content in the *Mmp13^Col2ER ^*mice (Figure 2A-B). The results of IHC revealed increased ColX in both the *Mmp13^Col2ER ^*and Cre-negative control groups following MLI, but the injury-associated induction was reduced in the *Mmp13^Col2ER ^*mice (Figure 2C). To investigate chondrocytes apoptosis, we performed TUNEL staining on knee joints eight weeks post-surgery in *Mmp13^Col2ER ^*and control mice. Results of TUNEL staining revealed that 28% of chondrocytes in articular cartilage underwent apoptosis in control mice that received MLI while only 6% of chondrocytes underwent apoptosis following MLI in *Mmp13^Col2ER ^*mice (Figure 2D and 2E).

#### CL82198 decelerates MLI-induced OA progression

**Figure 3:**
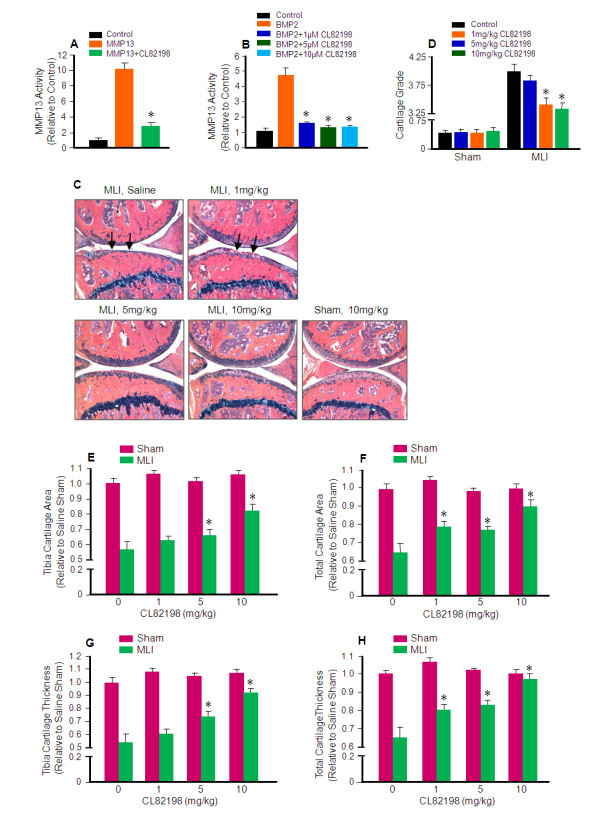
**CL82198 prevents and decelerates MLI-induced osteoarthritis progression**. (**A **and **B**) CL82198 inhibits matrix metalloproteinase (MMP13) activity *in vitro*. (**A**) Addition of 5 ng of MMP13, 5 ng of MMP13 and 10 μg/mL of CL82198, or substrate control into a 96-well plate. MMP13 enzyme activity was determined using the SensoLyte 520 MMP13 Assay Kit, and showed that CL82198 could block > 90% MMP13 activity *in vitro*. (**B**) Primary sternal chondrocytes were isolated from three-day-old wild type pups. Cells were plated in 12-well plates and treated with 100 ng bone morphogenetic protein 2 (BMP-2), 100 ng of BMP-2 and 1 μM of CL82198, 100 ng of BMP-2 and 5 μM of CL82198, 100 ng of BMP-2 and 10 μM of CL82198, or vehicle control for 60 hours. Cell culture media was collected and MMP13 activity was determined and showed that CL82198 could inhibit > 90% MMP13 activity produced by BMP-2-treated primary chondrocytes. (**C**) Inhibition of MMP13 by CL82198 protects against meniscal-ligamentous injury (MLI)-induced osteoarthritis (OA). MLI and sham surgeries were performed on 10-week-old wild type mice. CL82198 was administered to wild type mice one day after surgery by intraperitoneal injection every other day for 12 weeks at doses of 1, 5, or 10 mg/kg body weight. Normal saline was used as a control. Knee joints were collected, and sectioned 12 weeks post-surgery, and Alcian blue/Hematoxylin/Organge G staining was performed. (**D**) Histological grading by two blinded observers confirmed decreased articular cartilage degradation in mice treated with 1, 5, and 10 mg/kg of CL82198 compared to saline control mice (*P < 0.001). (**E**) The articular cartilage area at proximal tibiae was quantified by tracing the Alcian blue-positive area in the proximal tibia. Compared to saline MLI, tibial cartilage area was increased 9, 15, and 43% after treatment with 1, 5, and 10 mg/kg of CL82198, respectively (*P < 0.05). (**F**) Total articular cartilage area was quantified by tracing the Alcian blue-positive area in both the proximal tibia and the distal femur. Total cartilage area was increased 21%, 19%, and 38% with treatment of 1, 5, and 10 mg/kg of CL82198, respectively (*P < 0.05). (**G**) Tibia cartilage thickness was quantified by tracing the Alcian blue-positive thickness in the center of the tibial plateau. Compared to saline treatment, tibia cartilage thickness was increased 11%, 37%, and 70% with treatment of 1, 5, and 10 mg/kg of CL82198, respectively (*P < 0.05). (**H**) Total thickness was quantified by tracing the Alcian blue-positive thickness in both the proximal tibia and the distal femur. Total cartilage thickness was increased 23%, 27%, and 50% after injection of 1, 5, or 10 mg/kg CL82198, respectively (*P < 0.05).

We used CL82198, a selective MMP13 inhibitor, to determine if inhibition of MMP13 activity can alter the progression of OA-like degeneration in mice following MLI surgery. Addition of 10 μg/ml of CL82198 together with 5 ng active of MMP13 protein [17] blocked more than 90% of MMP13 activity *in vitro *(Figure 3A). In order to mimic the *in vivo *physiological situation more closely, we isolated primary sternal chondrocytes from three-day-old wild-type pups and treated the cells with bone morphogenetic protein 2 (BMP2) alone, or BMP2 plus 1, 5, or 10 μM CL82198 for 60 hours. MMP13 activity in the conditioned media was determined using an MMP13 assay kit. Results showed that after treatment with BMP2, MMP13 activity in conditioned media was significantly increased compared to vehicle control (Figure 3B). In contrast, treatment with 1 μM of CL82198 significantly inhibited MMP13 activity (84% inhibition). More than 90% of MMP13 activity was inhibited by treatment with 5 or 10 μM CL82198 (Figure 3B).

To investigate the efficacy of CL82198 in the inhibition of OA progression, we performed MLI surgery on 10-week-old WT mice, followed by i.p.injection of saline, 1, 5, or 10 mg/kg of CL82198 [18] every other day, beginning one day after surgery, for 12 weeks. Knee joints were harvested 12 weeks post-surgery. Histological results revealed that OA progression, most notably cartilage loss down to and even below the tidemark in saline control mice, was decelerated with 1, 5, or 10 mg/kg of CL82198 injection, particularly with the 10 mg/kg dose (Figure 3C). Cartilage grading results were consistent and treatment with 5 and 10 mg/kg doses of CL82198 had significantly lower scores than the saline control (Figure 3D). Histomorphometric analysis revealed that the articular cartilage area at the proximal tibiae was increased 9%, 15% and 43% (Figure 3E); total cartilage area was increased 21%, 19% and 38% (Figure 3F); articular cartilage thickness at the proximal tibiae was increased 11%, 37% and 70% (Figure 3G); and total cartilage thickness was increased 23%, 27% and 50% (Figure 3H) after injection of 1, 5, and 10 mg/kg of CL82198, respectively.

### CL82198 has protective effects on MLI-induced Col2 and proteoglycan loss and chondrocyte apoptosis

**Figure 4:**
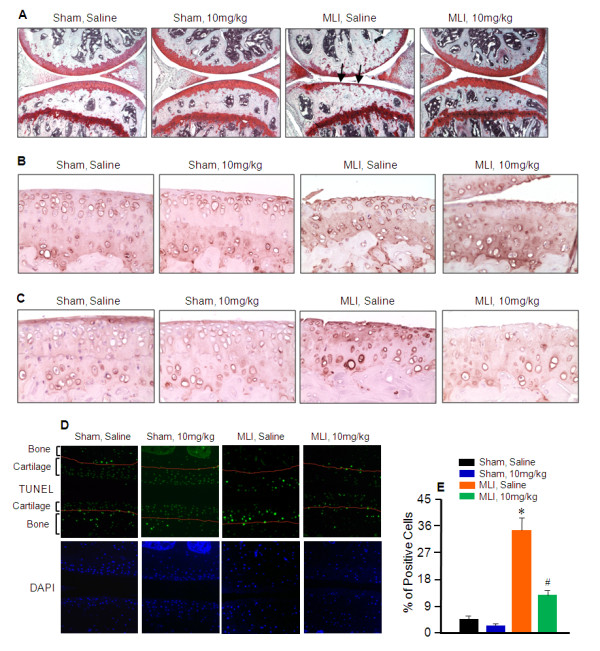
**CL82198 prevents and decelerates MLI-induced osteoarthritis progression**. Meniscal-ligamentous injury (MLI) and sham surgeries were performed on 10-week-old wild type mice. CL82198 was administered to wild type mice beginning one day after surgery by intraperitoneal injection every other day for 12 weeks at doses of 10 mg/kg body weight. Normal saline was used as a vehicle control. (**A**) Safranin O/Fast green staining was performed to assess proteoglycan content. Proteoglycan loss was reduced following CL82198 treatment. (**B**) Immunohistochemistry (IHC) of type II collagen (Col2) was performed to assess its protein level. Col2 loss was reduced following CL82198 treatment. (**C**) IHC of ColX was performed to assess its protein level. Type X collagen (ColX) induction was reduced following CL82198 treatment. (**D**) Terminal deoxynucleotidyl transferase dUTP nick end labeling (TUNEL) staining (in green) and 4',6-diamidino-2-phenylindole (DAPI) nuclear counterstaining (in blue) were performed to assess chondrocyte apoptosis. Chondrocyte apoptosis was reduced following CL82198 treatment. (**E**) Quantification of TUNEL staining positive cells was performed to assess chondrocyte apoptosis.

SO/FG staining was performed to assess proteoglycan content, and IHC was performed to evaluate Col2 and ColX protein levels in the mice with either saline or CL82198 (10 mg/kg) treatment. Results revealed greater proteoglycan and Col2 content in the CL82198 treatment group following MLI surgery compared to the saline treatment group (Figure 4A and 4B). Consistent with these findings, ColX levels were significantly decreased after CL82198 treatment compared to the saline control group (Figure 4C). To assess chondrocyte apoptosis, we performed TUNEL staining on samples from the saline and 10 mg/kg CL82198 treatment groups. Results of TUNEL staining demonstrated that 34% of chondrocytes underwent apoptosis in saline control mice following MLI, while only 15% of chondrocytes underwent apoptosis with CL82198 treatment (10 mg/kg) following MLI (Figure 4D and 4E).

## Discussion

OA is a degenerative joint disease and is the most prevalent form of arthritis. The major symptom of this disease is progressive cartilage break down and eventual complete loss of articular cartilage [1,19]. The pathogenesis of OA is not well understood, and there is currently no treatment to alter the progression of OA. In this study, we demonstrated that cartilage-specific deletion of *Mmp13 *decelerated the progression of OA. We also demonstrated that the MMP13 inhibitor CL82198 prevents OA progression in the MLI-induced murine knee OA model and may, therefore, represent a novel treatment option for OA patients to preserve articular cartilage and joint function instead of simply alleviating OA symptoms as existing treatments do.

Recently, Little *et al*. showed that global knockout of *Mmp13 *could prevent articular cartilage erosion [20]. However, there are several limitations related to this study. 1) The *Mmp13 *global knockout mice have a few abnormalities including focal regions of bony union in growth plates, tendency of metaphyseal flaring and increased trabecular bone mass that may affect OA study [21]. 2) OA is a chronic, progressive disease, and their study only assessed articular cartilage degradation four- and eight-weeks post-surgery-induced OA. 3) Their study relied on semi-quantitative histologic methods to characterize OA progression.

To understand better the role of MMP13 in OA development and progression, we generated inducible cartilage-specific *Mmp13^Col2ER ^*mice. Tamoxifen was administered when the mice were two-weeks-old, followed by MLI knee joint surgery to induce OA in *Mmp13^Col2ER ^*and Cre-negative control mice [22,12]. Our results demonstrate that while articular cartilage is nearly normal four weeks post-surgery in both groups, injured mice progressively develop an OA-like phenotype at the later 8-, 12-, and 16-week time points. The histomorphometric data showed that cartilage area and thickness at the proximal tibiae was significantly different at 12 and 16 weeks post-surgery in the *Mmp13^Col2ER ^*group compared to the control group. However, the total cartilage area and thickness only had a significant difference at 16 weeks post-surgery. This is likely because there was little evidence of cartilage degeneration or loss on the femoral condyle in either experimental group at 4, 8, 12, or 16 weeks post-surgery, which is consistent with previous studies using this model [20,12].

In addition to morphologic changes in articular cartilage structure, OA is also characterized by changes in matrix composition as well as changes in articular chondrocyte activity, including inappropriate levels of apoptosis [23-25]. Col2 and proteoglycan content were decreased in both *Mmp13^Col2ER ^*and control groups following MLI surgery. However, cartilage specific deletion of the *Mmp13 *gene partially prevented Col2 and proteoglycan loss. Deletion of the *Mmp13 *gene inhibited MLI-induced ColX expression as well. This finding is not unexpected as the cartilage is more intact in *Mmp13^Col2ER ^*mice compared to control mice at each time point. TUNEL staining revealed that articular chondrocytes underwent apoptosis in both the control and *Mmp13^Col2ER ^*group. However, deletion of the *Mmp13 *gene dramatically reduced articular chondrocyte apoptosis following MLI, potentially because the more intact cartilage in *Mmp13^Col2ER ^*mice may provide chondrocytes with environmental survival cues. Little *et al*. also showed increased articular chondrocyte apoptosis after surgery, but they did not identify a difference between the control and the *Mmp13 *KO groups. In their surgery model, they transected the medial meniscotibial ligament to destabilize the medial meniscus. In our surgery model, we transected the medial collateral ligament, detached the anterior horn of the medial meniscus from the tibial plateau, and created a tear. Thus, their surgical procedure may elicit a milder OA phenotype than our procedure, and this milder OA phenotype may be the reason for the apparent disparity in the apoptosis results in our and their studies.

Over the past 30 years, several MMP inhibitors have been developed as candidates for the treatment of arthritis, cancer and cardiovascular diseases [26]. However, most of these compounds have failed for a variety of reasons, including non-specificity and toxicity. Currently, no MMP inhibitor has been used in clinics [27]. Recently, Baragi *et al*. developed an MMP13 inhibitor, ALS 1-0635, and evaluated the efficacy of this compound in a rat OA model [28]. They gave ALS 1-0635 to rats twice-daily beginning one day before surgically-induced OA for three weeks, and found that ALS 1-0635 has chondro-protective effects. However, ALS 1-0635 only had an effect at a dose of 60 mg/kg. The large dose and frequent administrations of this compound suggest a relatively low specificity for ALS 1-0635 compound.

To explore the therapeutic potential of MMP13 inhibition for OA treatment, we investigated the ability of CL82198, a specific MMP13 inhibitor, to inhibit MMP13 activity *in vitro*. CL82198 is a chemical compound. Unlike ALS 1-0635 and other MMP13 inhibitors which exert their effects via metal chelation, CL82198 binds to the S1' pocket of MMP13 and showed no effects on MMP-1, -9, or TACE [17]. We found that CL82198 can block more than 90% of MMP13 activity when it reacts with active MMP13 directly. Since cartilage degeneration and articular chondrocyte dysfunction are hallmarks of OA, we isolated primary sternal chondrocytes from three-day-old WT pups to determine the effect of CL82198 on the activity of MMP13 secreted by chondrocytes undergoing hypertrophy. We treated sternal chondrocytes with BMP2 (100 ng/ml) for 60 hours to induce hypertrophy and MMP13 production/secretion. Meanwhile, the cells were also treated with CL82198 with BMP2 to inhibit BMP2-induced MMP13 activity. We found that CL82198 inhibited > 90% of MMP13 activity produced by BMP2-treated primary chondrocytes.

Next, we determined the efficacy of CL82198 *in vivo*. We performed MLI surgery on 10-week-old WT mice, followed by i.p. injection of saline or 1, 5, or 10 mg/kg of CL82198 [18] every other day, beginning one day after surgery. Histological data revealed that OA progression was decelerated following CL82198 administration, with the most pronounced effect at 10 mg/kg. Results of cartilage grading and histomorphometric analysis were consistent with histological data and treatment with CL82198 at 5 and 10 mg/kg doses resulted in significantly lower scores than the saline control.

Consistent with histological and histomorphometric findings, results from SO/FG staining and Col2 and ColX IHC revealed less proteoglycan and Col2 loss and a decreased ColX level in the CL82198 treatment group. Results of TUNEL staining indicate that about 20% fewer chondrocytes underwent apoptosis with treatment of CL82198 (10 mg/kg) following MLI compared to saline control mice. Taken together, these results suggest that OA progression is decelerated by the application of a MMP13 inhibitor.

Genetic manipulation is a powerful method to study the genetic causes of diseases and chemical compound delivery *in vivo *is a practical approach to test the efficacy of drug treatment. Here, we tested the role of MMP13 using *Mmp13 *cKO mice and using a MMP13 inhibitor in a surgically induced OA mouse model. Our studies suggest that inhibition of MMP13 expression or activity could efficiently prevent and decelerate OA progression. Our study provides initial evidence showing that a MMP13 inhibitor may have potential for the treatment of OA progression although future investigation is still required.

## Conclusions

Our study reveals that cartilage-specific deletion of the *Mmp13 *gene or inhibition of MMP13 activity decelerates the OA-like phenotype in a surgically induced OA model. Our studies also indicate that MMP13 inhibition could be used as a potential therapeutic strategy for prevention and treatment of OA.

## Abbreviations

AB/HO: Alcian Blue/Hematoxylin/Orange G; ADAMTS: a disintegrin and metalloproteinase with thrombospondin motifs; ANOVA: analysis of variance; BMP-2: bone morphogenetic protein 2; Col2: type II collagen; Col10: type X collagen; DAPI: 4',6-diamidino-2-phenylindole; ELISA: enzyme-linked immunosorbent assay; H & E: hematoxylin and eosin; IHC: immunohistochemistry; i.p.: intraperitoneal; MLI: meniscal-ligamentous injury; MMP: matrix metalloproteinase; *Mmp13^Col2ER^: Col2CreER;Mmp13^fx/fx^*; OA: osteoarthritis; SO/FG: Safranin O/Fast Green; TUNEL: terminal deoxynucleotidyl transferase dUTP nick end labelling; WT: wild type.

## Competing interests

The authors declare they have no competing interests.

## Authors' contributions

MW and ERS are responsible for study design, data acquisition and analysis and drafting and revising the manuscript. JL, HJ, and QHK are responsible for data acquisition and analysis. H-JI is responsible for data analysis and interpretation and revision of the manuscript. DC is responsible for study design, data analysis and interpretation and revision and final approval of the manuscript. All authors have read and approved the manuscript for publication.

## Supplementary Material

Additional file 1 **Deletion of the *Mmp13 *gene in chondrocytes at the postnatal stage has no significant effect on articular and growth plate cartilage morphology**. Histological sections from 10-week-old Cre-negative control and matrix metalloproteinase (MMP13) conditional knockout (cKO) mice (*Mmp13^Col2ER^*) treated with or without tamoxifen (tamoxifen was administered to two-week-old mice) were stained with Alcian blue/Hematoxylin/Orange G. No significant changes in articular and growth plate cartilage morphology were observed in these mice. Click here for file
