# Supplementary figures and images for: MMP13 is a critical target gene during the progression of osteoarthritis
Source: Arthritis Res Ther. 2013 Jan 8;15(1):R5. doi: 10.1186/ar4133 (PMC3672752; doi:10.1186/ar4133)

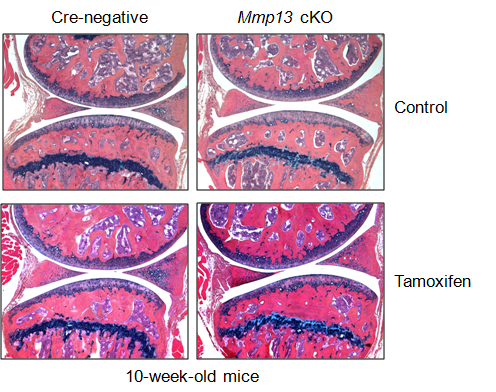

Supplement: Additional file 1 — Deletion of the Mmp13 gene in chondrocytes at the postnatal stage has no significant effect on articular and growth plate cartilage morphology. Histological sections from 10-week-old Cre-negative control and matrix metalloproteinase (MMP13) conditional knockout (cKO) mice (Mmp13Col2ER) treated with or without tamoxifen (tamoxifen was administered to two-week-old mice) were stained with Alcian blue/Hematoxylin/Orange G. No significant changes in articular and growth plate cartilage morphology were observed in these mice. [file ar4133-S1.TIFF]
